# Comparative Analgesic Efficacies of Ropivacaine and Bupivacaine for Postoperative Rectus Sheath Block in Paediatric Abdominal Surgery: A Meta-Analysis of Randomized Controlled Trial and Retrospective Cohort Studies

**DOI:** 10.1155/2021/5535730

**Published:** 2021-03-15

**Authors:** Lan Winnie, Yi-Hsuan Kao, Chien-Chang Liao, Takahiro Tamura, Ming-Long Chang, Kun-Yi Hsieh

**Affiliations:** ^1^Department of General Medicine, Taipei Medical University Shuang-Ho Hospital, New Taipei City, Taiwan; ^2^Department of General Medicine, Mackay Memorial Hospital, Taipei, Taiwan; ^3^Department of Anesthesiology, Taipei Medical University Hospital, Taipei 11031, Taiwan; ^4^School of Chinese Medicine, College of Chinese Medicine, Taiwan Medical University, Taichung, Taiwan; ^5^Research Center of Big Data and Meta-Analysis, Wan Fang Hospital, Taipei Medical University, Taipei, Taiwan; ^6^Department of Anesthesiology, Nagoya University Graduate Institute of Medicine, Nagoya, Japan; ^7^Department of Emergency Medicine, Taipei Medical University Hospital, Taipei 11031, Taiwan; ^8^Emergency Department, Taipei Municipal Wanfang Hospital, Taipei 116, Taiwan

## Abstract

**Background:**

The optimal dose and concentration of analgesic efficacy of ropivacaine (RPV) and bupivacaine (BPV) for postoperative pain relief in paediatric abdominal surgery patients is still unclear. Therefore, this meta-analysis compared the efficacy of these analgesics, their administered modes (ultrasound-guided RSB versus LAI) for postoperative pain relief, and side effects.

**Methods:**

Three databases, PubMed, Embase, and Cochrane Database of Systematic Reviews, were exhaustively searched with predefined keywords. Eight randomized clinical trials and retrospective studies were selected. Analgesic effect, postoperative pain score, level of side effect, applied dose, and concentration of drug were analysed.

**Results:**

Drug dose ranged from 0.5–2.5 mL/kg of 0.2 to 0.5% concentrations. Male participant for RSB and LAI treatment groups varied from 40–62% and 25–83%, respectively. Mean age of RSB and LAI groups ranged from 3.8–11.65 years and 4.3–11.27 years, respectively. Our meta-analysis revealed that RSB could reduce total opioid use postoperatively (WMD = −0.02, 95% CI: −0.02, −0.02), with *I*^2^ value of 15%. We found that the RPV (0.25%, 2.5 ml/kg) was optimal in suppressing the pain. Its lower concentration (0.2%) was ineffective, whereas higher one (0.375%) seems to increase risk of systemic toxicity. Similarly, BPV (0.25%, 2.5 mg/kg) efficaciously reduced the pain score, while its lower concentration was ineffective. The combined postoperative pain score in the RPV-treated group was found to be significantly reduced (*p* < 0.01) with *I*^2^ value of 85% indicating high heterogeneity.

**Conclusion:**

Both RPV and BPV were significantly effective in reducing postoperative pain score. It appears that RSB could be a preferred choice to deliver analgesia, due to reduced opiate dose requirement and improved clinical safety without significant postoperative adverse events.

## 1. Introduction

Over 80% of patients experience acute postoperative pain, the management of which still remains a significant problem for anaesthesiologists [1]. Moreover, pain severity assessment, and its management among paediatric patients in particular, is a matter of great concern. Though the postoperative pain in more than two-thirds of children can usually be well controlled with opiates [2], these have been found associated with various adverse events, of which the most commonly reported are nausea, vomiting (40%), pruritus (20–60%), constipation (15–90%), and even some life-threatening respiratory depression (0.0013%) [3]. Hence, regional anaesthesia is the preferred alternative for paediatric patients, owing to its improved recovery proven safety [4]. However, the use of local anaesthetics is also related to drug toxicity in children [5] due to reduced (20% to 40%) levels of *α*−1 acid glycoprotein (AAG) in plasma in neonates compared to normal adult values.

Among regional anaesthetic procedures, rectus sheath block (RSB) has been reported as an effective pain relief for umbilical or other midline surgical incisions. The process anaesthetizes the anterior rami of nerves T9–11 [6]. It was first described in 1899 and later applied on paediatric umbilical hernia repair in mid-1990 [7]. Later, the technique has evolved to incorporate ultrasound guidance to identify the anatomy and reduced complications for better safety, accuracy, and rapidity. Notably, RSB can not only suppress excessive stimulation caused by skin incision, but also reduce the amount of anaesthetic agent used during surgery (including opioids) as well as postoperative pain [8].

Ropivacaine (RPV) and bupivacaine (BPV) are the commonly used opioid-sparing analgesics that provide long-lasting pain control through RSB. Both of these drugs are structurally similar and employed as local anaesthetics in caudal block for perioperative pain relief after perineal and lower abdominal surgeries. Compared with BPV, the RPV has been reported to produce fewer side effects in the cardiovascular as well as central nervous system [9]. To date, both medications have commonly been used in various hospitals as analgesic agent for RSB; however, no specific guidelines have been suggested for their choice or dosage. A few published randomized controlled trials have investigated the efficacy of RPV or BPV along the surgical wound; however, their role in RSB in paediatric patients has been inconclusive. Therefore, we performed this meta-analysis to evaluate the comparative efficacy of RPV and BPV in the management of postoperative pain after RSB in paediatric patients. Specifically, this systematic study aims toward finding the best concentration and dosage that is used for postoperative RSB using RPV and BPV, since paediatric anaesthetics should be used with extreme precaution.

## 2. Materials and Methods

We registered the protocol on the International Prospective Register of Systematic Reviews (PROSPERO) with registration number CRD42020162719.

### 2.1. Literature Selection Criteria

**Figure 1:**
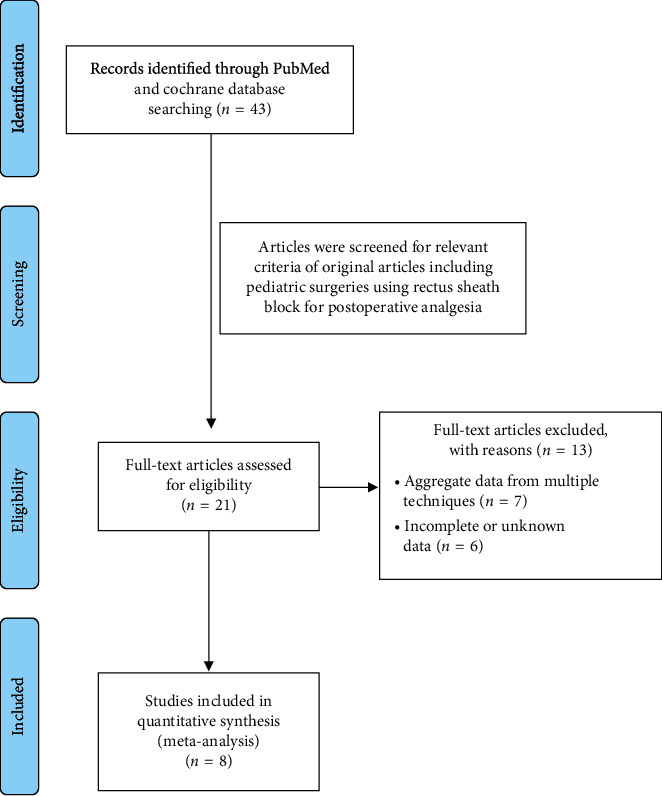
PRISMA flow diagram showing literature search and selection of studies in the analysis.

Preferred reporting items for systemic meta-analyses (PRISMA) protocol was followed for study data collection and their analysis (Figure 1). The PubMed, Embase, and the Cochrane Database of Systematic Reviews were exhaustively searched using the following keywords: rectus sheath block, children, paediatric surgery, analgesia, and concentration for studies conducted between June 2006 and November 2019. Additional relevant studies were included through inspecting the primary shortlisted research; however, the search was limited to only published research and not to unpublished works. To achieve our aims, we selected paediatric patients who underwent abdominal surgery as participants (P), the anaesthetic agents (RPV and BPV), and their various dosage administered through ultrasound-guided RSB as interventions (I), compared to LAI (C), the results of which were evaluated on the basis of pain score, total opiate use, and side effects (O).

### 2.2. Inclusion and Exclusion Criteria

Only randomized control trials (RCTs) and retrospective studies were included for further selection. Two researchers (W. L. and Y. H. K.) independently screened all titles and abstracts from the studies identified using the above search terms and read the full text to further screen and evaluated the studies according to inclusion and exclusion criteria. The following criteria were used for inclusion of the study for further meta-analysis: (i) only paediatric patients with the age lower than 18 years who received abdominal surgery were included; (ii) different concentrations of RPV or BPV in RSB were used as test for postoperative analgesia and local anaesthetic infiltration (LAI) was considered as control; (iii) Possibility to extract valid data from the study; and (iv) studies were published in English language. In addition, studies with control group other than LAI were excluded from the analysis.

### 2.3. Data Extraction

The following data points such as first author, publication of year, type of surgery, number of RSB and control groups, choice of analgesia, intervention measures, local analgesia, dosage and concentration, identification of opioid consumption within 24 hours postsurgery (consumption is converted in mg/kg), and assessment of pain score immediately after surgery were included. Moreover, symptoms of side effects such as postoperative nausea, vomiting, and other related morbidities were also recorded 24 hours postsurgical intervention. The data from relevant and included studies were extracted by W. L. and Y. H. K. Further, any discrepancies were resolved by discussion between W. L. and Y. H. K.

### 2.4. Evaluating the Risk of Biasness

Risk of biasness in the included studies incorporate the following possible Cochrane-based biasness features: possible selection biasness (sequence generation and allocation concealment), possible performance and detection biasness (blinding of participants, personals, and outcomes assessment), possible attrition biasness due to withdrawals (incomplete outcome data), selective reporting biasness, and other sources of biasness were assessed by W. L. and Y. H. K. The risk of biasness was characterized in three categories, i.e., low, high, and unclear risk of bias, which were represented through colour coding of green, red, and yellow, respectively.

### 2.5. Statistical Analysis

Meta-analyses were performed with the assistance of RStudio to generate linear random effect models of the relationship between RSB and LAI through forest plots. The effect of RPV and BPV concentration was assessed in terms of mean values of postoperative pain scores, the 24-hour opioid consumption, and the incidence of postoperative side effects, i.e., nausea and vomiting. The data have been reported in mean and standard deviation (SD) and interquartile range (IQR), using Wan et al.'s method using the following formula:

(1) $$\begin{array}{c} \frac{q_{1}+m+q_{3}}{C}, \end{array}$$

where *q*_1_ and *q*_3_ represent first and third quartile, respectively, whereas *m* signifies median.

Cochran's *Q* test was used to evaluate the statistical heterogeneity. Further, Higgins's *I*^2^ statistics was used to observe the proportion of variation and inconsistency of treatment effect among the studies. Statistical significance was set to *p* ≤ 0.01 for the Cochrane *Q* test. Significant heterogeneity between studies was assessed by *I*^2^ tests, where *I*^2^ < 30%, 30–60%, and >60% suggests a “low,” “moderate,” and “high” level of heterogeneity, respectively.

## 3. Results

### 3.1. Characteristics of the Trials

**Figure 2:**
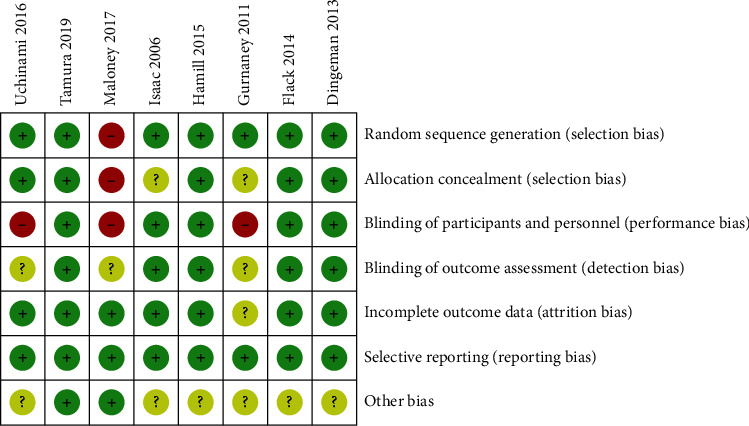
Evaluation summary of bias risk items for each included study. The green, red, and yellow circles represent low, high, and unclear risk of bias, respectively.

The literature searches with keywords hit 43 articles published in English; of these, 21 full articles were assessed for further screening. However, after introducing screening criteria, only 8 studies were found to be eligible for further analysis [10–17]. The remaining studies were excluded due to aggregate data from multiple techniques and lack of desired data. These selected studies represented a total of 672 patients. Of these, 275 and 397 patients were part of two retrospective studies and RCT, respectively. Further, these trials were conducted from 2006 to 2019 and compared with rectal sheath block (RSB) administered with the local anaesthesia infiltration (LAI). The included studies compared RPV and BPV efficacy in managing postoperative pain through RSB-mediated delivery in children. In addition, the studies compared RSB with LAI. Further, biasness of this study was evaluated through Cochrane risk of bias tool, and our assessment indicated a low risk of bias in the majority of trials (Figure 2).

Table 1 summarizes the traits of trials included for meta-analysis. The numbers and age of patients ranged from 13–275 and 1–17 years, respectively. Out of 8 trials, 7 were RCTs [10–13, 16, 17], one was pilot study [14], and another one was retrospective [15]. Dingeman et al., Gurnaney et al., Fleak et al, Tamura et al., Hamil et al., and Uchinami et al. [10–13, 16, 17] conducted trials for paediatric umbilical hernia repair, whereas Maloney et al. [15] and Isaac et al. [14] studied the potency of specified drugs for paediatric appendectomy. The drug dose ranged from 0.5–2.5 mL/Kg of 0.2 to 0.5% concentrations. Male participant for RSB and LAI treatment groups varied from 40–62% and 25–83%, respectively. Further, in the included studies, the mean age of RSB groups ranged from 3.8 to 11.65 years, whereas that of LAI groups varied between 4.3 and 11.27 years.

### 3.2. Primary Outcomes

#### 3.2.1. Total Morphine (Opioid) Consumptions

**Figure 3:**
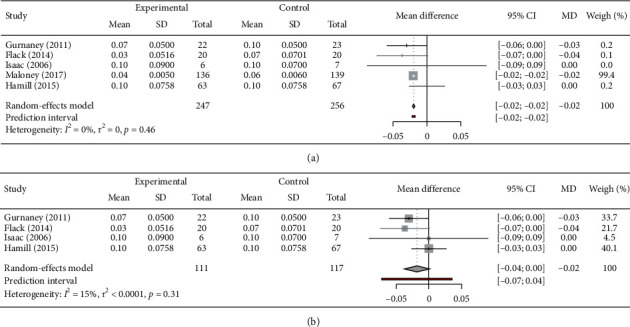
Primary outcomes. Forest plot with effect size, estimated heterogeneity (*I*^2^ and *τ*^2^) and confidence intervals (CIs) for meta-analysis evaluating total morphine consumption in the postoperative anaesthetic agents, RPV and BPV rectus sheath block of all groups, the effect of BPV and RPV on postoperative pain (a) and (b) except Maloney's study. The column “Mean” represents the mean dosage of opioid use (mg/kg), while “Total” stands for the patient number included in the studies. The square symbol on forest plot denotes the weighted mean difference for individual studies, with 95% CI of the difference represented as a solid line. The size of square and thickness of the 95% CI line resemble sample size. WMD: weighted mean difference; RPV: ropivacaine; BPV: bupivacaine.

Gurnaney (2011), Flack (2014), Isaac (2006), Maloney (2017), and Hamill (2015) provided participant data on total opiate consumption: three in umbilical hernia repair and two in appendectomy. All studies reported total opioid consumption as mean (SD) or median interquartile range (IQR). Our meta-analysis confirmed the findings that RSB can reduce total opioid use postoperatively (WMD = −0.02; 95% CI: −0.02–.02) (Figure 3(a)). Moreover, an *I*^2^ value of 0 indicated no observed heterogeneity. Additionally, one study by Maloney (2017) reported a very high WMD of 99.4%, indicating high risk of biasness. To overcome this single study-based risk of bias, the study of Maloney (2017) was excluded and the result is presented as a forest plot in Figure 3(b), which reveals an *I*^2^ of 15% indicating the minimum heterogeneity. However, in this group, reduction in opioid consumption was found insignificant (*p* < 0.05). Moreover, in both the RSB and LAI approaches, the studies of Isaac (2006) and Hamill (2015) showed no difference in postoperative consumption of opioid.

#### 3.2.2. Pain Score

**Figure 4:**
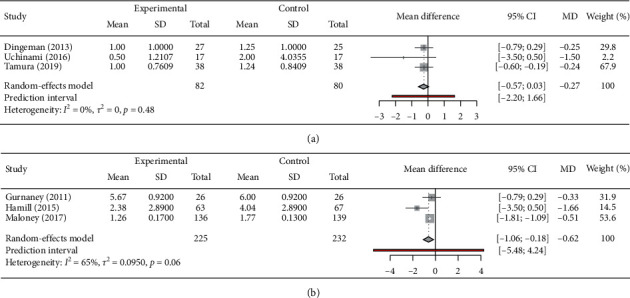
Pain score of (a) RPV group and (b) BPV group. The square symbol on forest plot denotes the weighted mean difference for individual studies, with 95% CI of the difference represented as a solid line. The size of square and thickness of the 95% CI line resemble sample size. RPV: ropivacaine; BPV: bupivacaine.

We had six studies providing pain score, of which 3 used BPV (Gurnaney (2011), Hamill (2015), and Maloney (2017)) and 3 used RPV (Dingeman (2013), Yuka Uchinami (2016) and Takahiro Tamura (2019)). Four studies reported pain score on FACES (Gurnaney (2011), Maloney (2017), Dingeman (2013), and Takahiro Tamura (2019)), two on the face, legs, activity, cry, and consolability (FLACC) (Yuka Uchinami (2016) and Takahiro Tamura (2019)), one on FPS-R (Hamill (2015)), and one on VAS (Maloney (2017)). Mean (SD) in RPV (Figure 4(a)) and BPV-treated group (Figure 4(b)) was calculated as −0.97 (95% CI: −0.57; 0.03) and 0.62 (95% CI: −1.06; −0.18), respectively.

**Figure 5:**
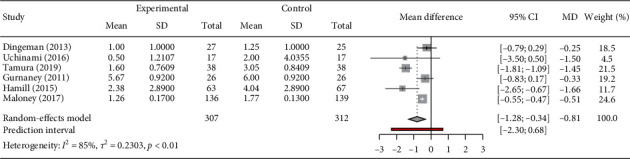
Pain score of all studies. The pain score was measured on the numerical scale of 0–10. The square symbol on forest plot denotes the weighted mean difference for individual studies, with 95% CI of the difference represented as a solid line. The size of square and thickness of the 95% CI line resemble sample size.

The combined postoperative pain score in RPV-treated group was found to be significantly reduced (*p* < 0.01); however, the *I*^2^ value of 85% indicates high heterogeneity (Figure 5). In addition, the doses of RPV used in various combinations include 0.5 ml/kg of 0.2% in Dingeman (2013), 0.4 ml/kg of 0.375% Uchinami (2016), and 0.5 ml/kg of 0.25% in Tamura's. The analysis of this group indicated considerable pain reduction in the trials of Dingeman (2013) and Uchinami (2016), though not significant. However, the trial of Tamura (2019) reported significant reduction in the pain score (MD: −1.45 and CI: −1.81–1.09) in comparison to control group. Similarly, RPV also showed considerable reduction in combined pain score (MD: −0.62, 95% CI: −1.06; −0.18), and I^2^ score of the combined studies of Gurnaney (2011), Hamill (2015), and Maloney (2017) was found to be 65% implying higher heterogeneity. Further, the individual trail of Gurnaney (2011) found no significant reduction in pain score (MD: −0.33, 95% CI: −0.83–0.17). On the contrary, the trials of Hamill (2015) and Maloney (2017) reported significant postoperative pain score reduction. Notably, the combined effect of RPV and BPV treatment was found to be successful in significantly reducing pain score (MD: −1.84, 95% CI: −1.28–0.34 *p* < 0.01). However, the heterogeneity *I*^2^ score of 85% indicates high heterogeneity.

### 3.3. Secondary Outcome: Side Effects

Four studies reported the adverse events among RSB and LAI groups such as nausea and vomiting during treatment [12, 14, 16, 17] (Table 2). Nausea and vomiting were greater in Gurnaney (2011) study, whereas no case was reported in the RSB group of Isaac (2006) study. The equal number of postoperative nausea and vomiting was found in both RSB and LAI groups of Tamura's and Uchinami's studies. Moreover, the other remaining five studies did not report any side effects in any groups. Notably, these complications did not significantly differ among the groups.

## 4. Discussion

To the best of our knowledge, this is the first meta-analysis on comparative analgesic efficacies of RPV and BPV for postoperative paediatric anaesthesia. Further, we have also assessed the optimal dose and delivery modes (RSB and LAI) to evaluate the pain relief efficacy and safety. These drugs are employed postoperatively through RSB or LAI to lower pain for paediatric umbilical hernia repair and appendectomy [10]. The paediatric patients generally have a higher risk of cardiovascular and pulmonary morbidity and mortality when compared with adults [18]. Neurotoxicity concerns also appear greatest in infants with prolonged or repeated anaesthetic exposures, which could be reduced by regional anaesthesia leading to improved postoperative outcomes. Therefore, based on 8 selected studies including 672 paediatric patients [10–17], we determined the comparative efficacy of RPV and BPV in terms of dose, concentration, and safety among children (1–17 years). Our findings revealed that 0.25% concentration of RPV was optimal in lowering the pain 24 hours after surgery. The lower concentration (0.2%) was found to be ineffective, whereas the higher concentration (0.375%) seemed to increase the risk of systemic toxicity. However, most of the included studies employed 0.25% concentration of BPV and the dose of 2.5 mg/kg which was efficacious in reducing the pain score, implying that the higher dose of BPV needs to be carefully considered for pain control among paediatric patients postabdominal surgery. Previously, a meta-analysis of 6 randomized blind trials including 403 cases compared the efficacy of RPV and BPV (2.5 mg/ml) pain in labour on neonatal outcome and mode of delivery [19]. The study reported a similar pain relief and consumption of the two drugs among neonates 2 h postdelivery. However, after 24 hours, RPV represented higher number of neonatal with neurologic and adaptive capacity score (NACS) of 35 compared to BPV. This has been attributed to lower lipid solubility and a shorter terminal half-life of RPV than BPV. Thus, one may hypothesize that BPV persists in the neonatal neural tissues longer than RPV, such that its subtle changes on neurological function become evident at a time when the effects of RPV have declined. In line with our findings, a randomized clinical trial revealed an equivalent pharmacological action of RPV (0.5%) to BPV (0.5%) for maxillary lateral incisor infiltrations [20]. Besides, in a systemic review of fifteen research articles including 381 patients, it has been evidenced that RSB is a potential effective morphine delivery target to reduce its requirements and effectivity in pain control postabdominal surgery [21]. The study documented that local anaesthetic in RSB could result in detectable systemic concentrations that may exceed commonly accepted thresholds of LA systemic toxicity. Moreover, a clinical evidence-based clinical update suggested no difference in efficacy between RPV and BPV in paediatric caudal anaesthesia with no adverse events [22]. However, compared to RPV, BPV was found to be more effective in motor block.

## 5. Conclusions

Taken together, we found that both RPV and BPV were effective in significant reduction of postoperative pain score in paediatric patients. We also conclude that, compared to optimal concentration and dose of RPV (0.25%, 2.5 ml/kg), the lower or higher dose either was ineffective or may increase the risk of systemic toxicity, whereas lower concentration of BPV was ineffective compared to optimal dose (0.25%, 2.5 mg/kg) in reducing pain. Hence, its higher doses are suggested. The delivery of opioids through RSB could not only reduce their consumption for pain relief, but also reduce the risk of systemic toxicity. However, the limited clinical trials, lack of specific guidelines for RSB, and opioid use among paediatrics limit the application of the findings of this study at large scale limits.

## Figures and Tables

**Table 1:** Study characteristics included in analysis.

Author and publication year	Study design	Characteristics N (RSB/control)	Gender (M%, RSB/LAI)	Age (mean, RSB/LAI)	Control group	Surgery	Local analgesic	Analgesic conc.	Pain score	Adverse effect with opioid (PONV)
Isaac and colleagues [14]	Pilot study	13 (7 : 6)	43%; 83%	5.9; 3.9	LAI	Umbilical hernia repair	BPV	0.8 ml/kg of 0.25%	CHEOPS	0 : 01
Gurnaney and colleagues [12]	Prospective randomized observer-blinded	52 (26 : 26)	54%; 56%	7.7; 7.9	LAI	Umbilical hernia repair	BPV	1.5 mg/kg of 0.25%	FACES	4 : 02
Dingeman and colleagues [10]	Prospective RCT	52 (27 : 25)	44%; 56%	6.0; 6.1	LAI	Umbilical hernia repair	RPV	0.5 mL/kg of 0.2%	FACES	NA
Flack and colleagues [11]	Randomized blinded	40 (20 : 20)	40%; 25%	5.85; 4.95	LAI	Umbilical hernia repair	BPV	0.5 mL/kg of 0.25%	FLACC 1–3 y/o, FACES 4–17 y/o	NA
Hamill and colleagues [13]	RCT	130 (63 : 67)	59%; 67%	5.3; 5.3	Normal saline	Appendectomy	BPV	2.5 mg/kg of 0.25%	FPS-R	NA
Uchinami and colleagues [17]	Prospective randomized trial	34 (17 : 17)	59%; 59%	3.8; 4.3	LAI	Laparoscopic surgery	RPV	0.4 ml/kg of 0.375%	FLACC	2 : 02
Maloney and colleagues [15]	Retrospective analysis	275 (136 : 139)	62%; 60%	11.65; 11.27	LAI	Trans-umbilical laparotomy-assisted appendectomy	BPV	0.25%/0.5%; total of 1 ml/kg/0.5 ml/kg	FACES < 12 y/o; VAS > 12 y/o	NA
Tamura and colleagues [16]	RCT	76 (38 : 38)	47%; 42%	5.03; 4.92	LAI	Inguinal hernia repair	RPV	0.5 ml/kg of 0.25%	FLACC/FS	1 : 01

RSB: rectal sheath block; LAI: local anaesthetic infiltration.

**Table 2:** Side effects.

Author and publication year	RSB no.	RSB SE	LA no.	LA SE
Isaac and colleagues [14]	7	0	6	1 (nausea)
Gurnaney and colleagues [12]	26	4 (2N + 2 V)	26	2 (1N + 1 V)
Dingeman and colleagues [10]	27	NA	25	NA
Flack and colleagues [11]	20	NA	20	NA
Hamill and colleagues [13]	63	NA	67	NA
Uchinami and colleagues [17]	17	2 (PONV)	17	2 (PONV)
Maloney and colleagues [15]	136	NA	139	NA
Tamura and colleagues [16]	38	1 (PONV)	38	1 (PONV)

RSB: rectal sheath block no.; SE: side effect; LA: local anaesthesia infiltration; NA: not applicable; N: nausea; V: vomiting; PONV: postoperative nausea and vomiting

## Data Availability

The data used to support the findings of this study are available from the corresponding author upon request.
